# Supplementary material for: Good Vibrations: Structural Remodeling of Maturing Yeast Pre-40S Ribosomal Particles Followed by Cryo-Electron Microscopy
Source: Molecules. 2020 Mar 3;25(5):1125. doi: 10.3390/molecules25051125 (PMC7179242; doi:10.3390/molecules25051125)
Supplement: Supplementary file 1 [file molecules-25-01125-s001.pdf]

Supplementary Figure 1

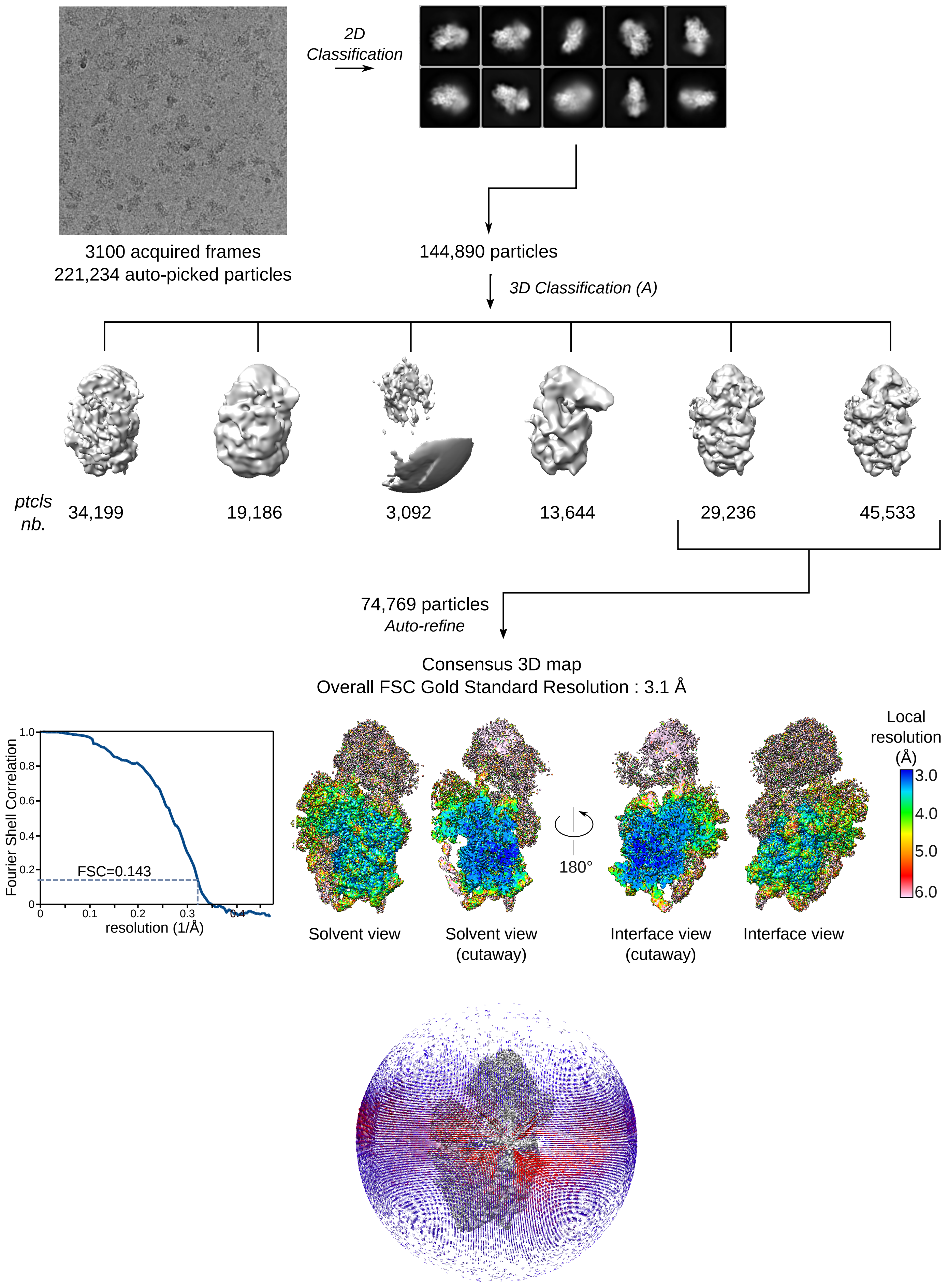

Supplementary Figure 2

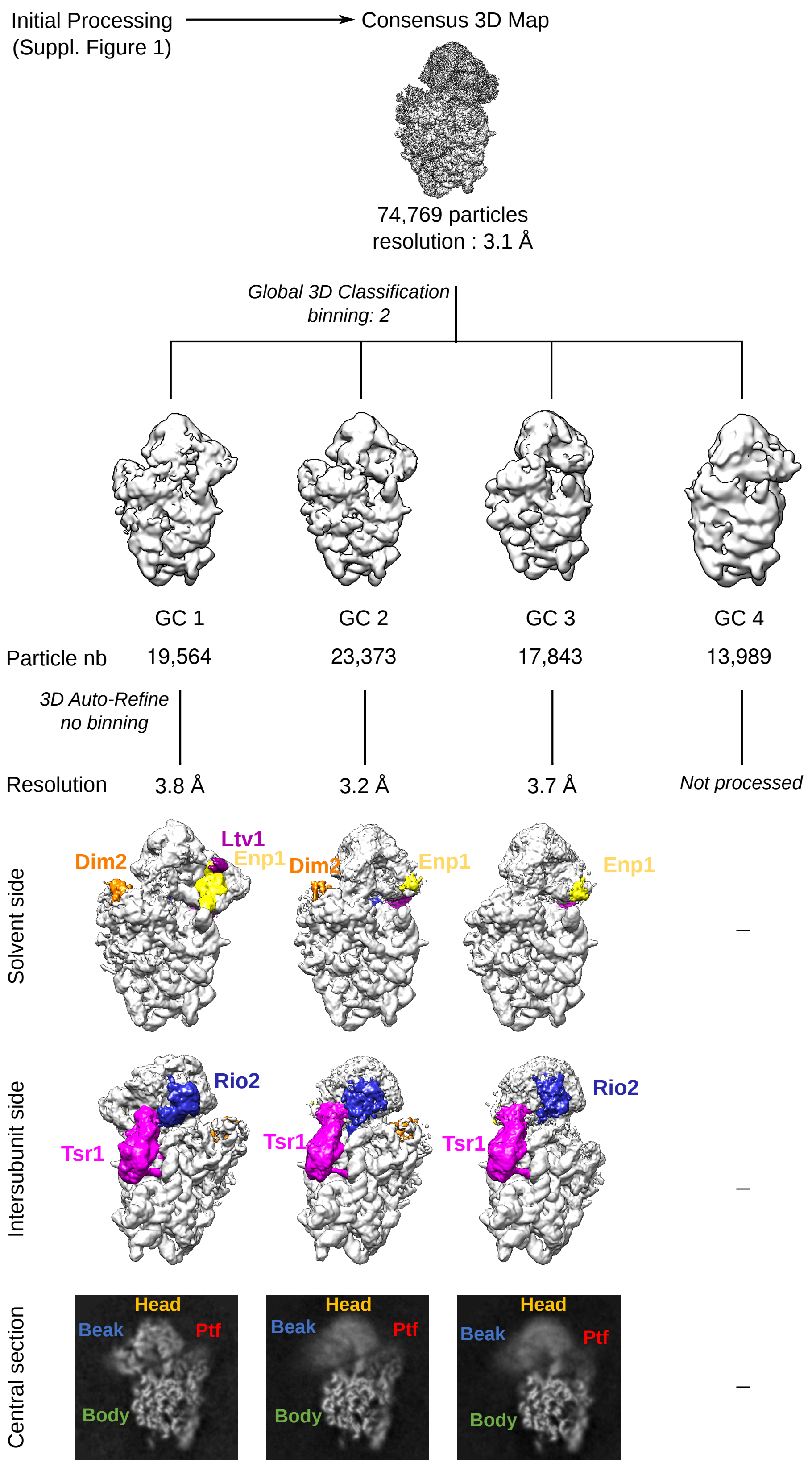

Supplementary Figure 3

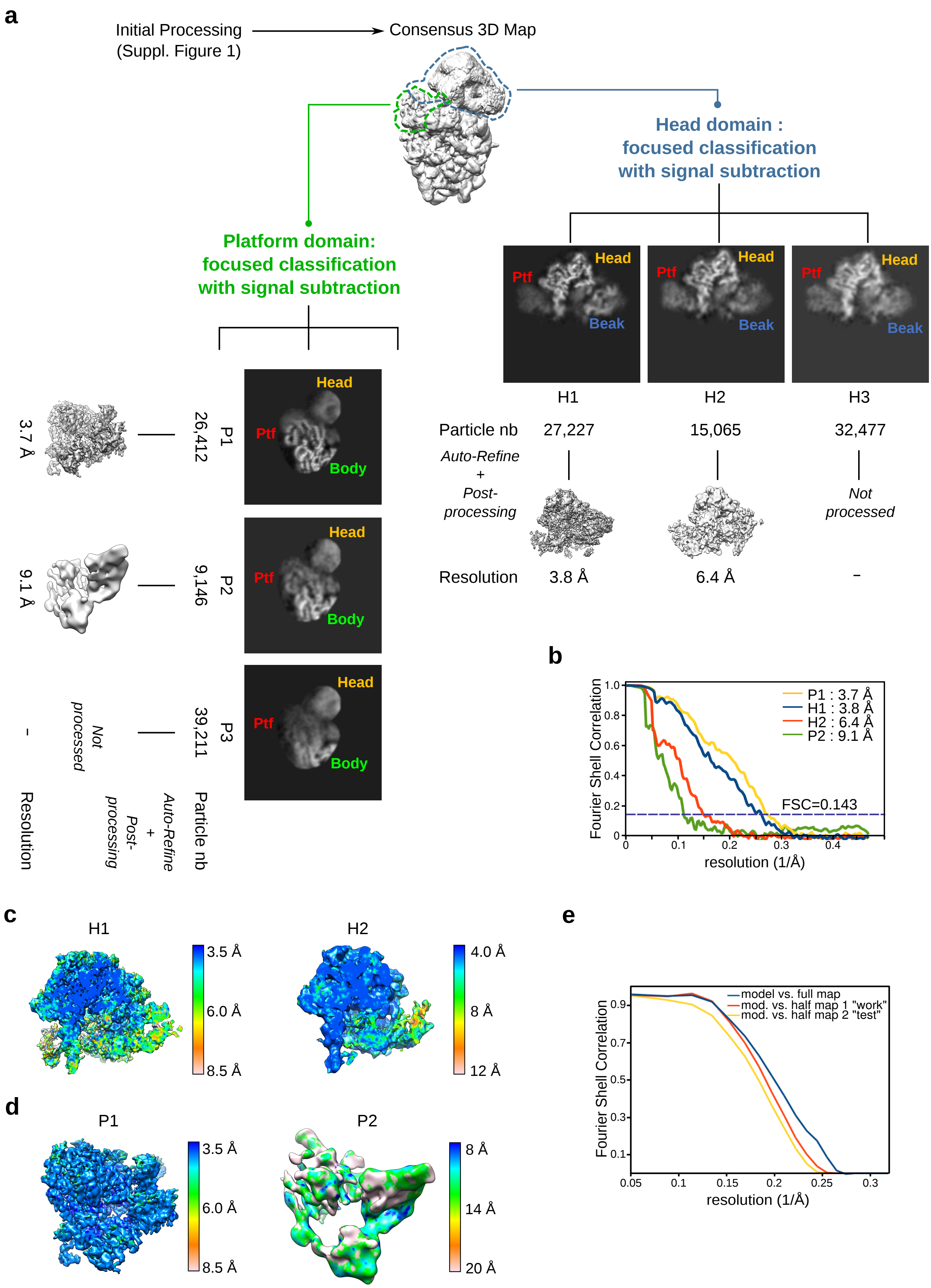

**EM Data collection**							
Microscope model	FEI Titan Krios cryo-transmission electron microscope						
Detector model	Gatan K2 summit direct electron detector						
Number of datasets	1						
Number of micrographs collected	3,100						
Pixel size (Å)	1.067						
Defocus range (µm)	0.8– 2,8						
Voltage (kV)	300						
Electron dose ($e^{-}Å^{-2}$)	29.4						
Name of 3D reconstruction / model	Early cytoplasmic Tsr1-FPZ	Consensus Tsr1-FPZ	GC1 Tsr1-FPZ	H1 Tsr1-FPZ	H2 Tsr1-FPZ	P1 Tsr1-FPZ	P2 Tsr1-FPZ
EMDB entry of map		EMD-10715	EMD-10713	EMD-10716	EMD-10717	EMD-10718	EMD-10719
PDB entry of the full model	6Y7C						
Final number of particles		74,769	19,564	27,227	15,065	26,412	9,146
Resolution (Å) (FSC threshold = 0.143)		3.12	3.80	3.84	6.40	3.69	9.14
Map sharpening B-factor ($Å^2$)		-147	-157	-172	-326	-165	-303
**Refinement and model validation statistics**[(a)]							
Model refinement resolution range (Å)<br><br>Model resolution (Å) (FSC threshold = 0.143)<br>Clashscore (all atoms)	20-3.2<br><br><br>3.9<br><br>9.98						
MolProbity Score<br><br><br>Protein	2.17						
Rotamer outliers (%)	1.0						
Rmsd (bonds lengths, Å)	0.01						
Rmsd (angles, °)	1.18						
Ramachandran plot (%)<br>favored	 <br>85.36						
allowed	14.32						
outliers	0.32						
RNA							
Correct sugar puckers (%)	98.4						
Good backbone conformation (%)	64.0						

[a]Models were validated using MolProbity implemented in PHENIX.REFINE (Adams et al., 2010)

Supplementary Table 1. Cryo-EM data collection, atomic models refinemement and validation statistics.
